# Mixed Maximum Loss Design for Optic Disc and Optic Cup Segmentation with Deep Learning from Imbalanced Samples

**DOI:** 10.3390/s19204401

**Published:** 2019-10-11

**Authors:** Yong-li Xu, Shuai Lu, Han-xiong Li, Rui-rui Li

**Affiliations:** 1 Department of Mathematics, Beijing University of Chemical Technology, Beijing 100029, China; xuyongli2312@sina.com (Y.-l.X.); lushuaie@gmail.com (S.L.); 2 State Key Laboratory of High Performance Complex Manufacturing, Central South University, Changsha 410083, China; 3 Department of Systems Engineering and Engineering Management, City University of Hong Kong, Hong Kong 999077, China; mehxli@cityu.edu.hk; 4 College of Information Science & Technology, Beijing University of Chemical Technology, Beijing 100029, China

**Keywords:** convolutional neural network, mixed maximum loss minimization, optic disc segmentation, optic cup segmentation, glaucoma screening

## Abstract

Glaucoma is a serious eye disease that can cause permanent blindness and is difficult to diagnose early. Optic disc (OD) and optic cup (OC) play a pivotal role in the screening of glaucoma. Therefore, accurate segmentation of OD and OC from fundus images is a key task in the automatic screening of glaucoma. In this paper, we designed a U-shaped convolutional neural network with multi-scale input and multi-kernel modules (MSMKU) for OD and OC segmentation. Such a design gives MSMKU a rich receptive field and is able to effectively represent multi-scale features. In addition, we designed a mixed maximum loss minimization learning strategy (MMLM) for training the proposed MSMKU. This training strategy can adaptively sort the samples by the loss function and re-weight the samples through data enhancement, thereby synchronously improving the prediction performance of all samples. Experiments show that the proposed method has obtained a state-of-the-art breakthrough result for OD and OC segmentation on the RIM-ONE-V3 and DRISHTI-GS datasets. At the same time, the proposed method achieved satisfactory glaucoma screening performance on the RIM-ONE-V3 and DRISHTI-GS datasets. On datasets with an imbalanced distribution between typical and rare sample images, the proposed method obtained a higher accuracy than existing deep learning methods.

## 1. Introduction

Glaucoma is an irreversible neurodegenerative ophthalmic disease as well as the second leading cause of blindness in the world. By 2020, the number of glaucoma patients will reach about 80 million worldwide and this number will increase to 110 million by 2040 [1]. Patients with early glaucoma usually have no obvious symptoms. As a result, a large proportion of patients are not aware of the disease until unrecoverable visual loss occurs. Hence, early detection and treatment of glaucoma are important for vision protection.

**Figure 1:**
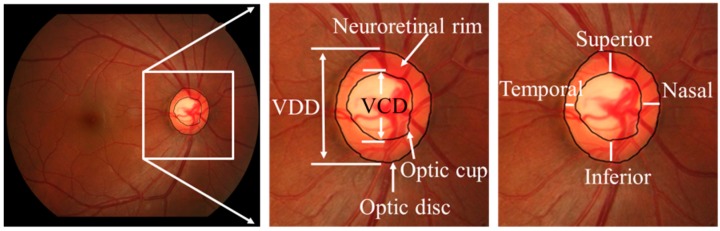
Optic disc, optic cup and neuroretinal rim in a fundus image.

Fundus photography is the most commonly used method for diagnosing glaucoma. For the diagnosis of glaucoma, the most important structures in a fundus image are the optic disc (OD) and optic cup (OC). The optic disc is the visible part of the optic nerve from which the nerve fibers leave the eyes. In Figure 1, the central depression of the optic disc is known as the optic cup and the area around the optic cup is known as the neuroretinal rim. Based on OD and OC, ophthalmologists can use some indicators such as vertical cup-to-disc ratio (VCDR) and the “inferior, superior, nasal, and temporal” (ISNT) rule to make a diagnosis [2]. The VCDR is defined as the ratio of the vertical diameter of the optic cup (VCD) to the vertical diameter of the optic disc (VDD). The ISNT rule refers to the fact that in a normal eye, the inferior rim is usually the thickest part, then the superior rim, followed by the nasal rim, with the temporal rim being the thinnest part.

However, in order to accurately locate OD and OC, ophthalmologists need to have a wealth of clinical experience. For primary ophthalmologists, accurately and efficiently determining the location of OD and OC in fundus images is a challenging task. Therefore, high-precision automatic extraction of OD and OC is of great significance for the diagnosis of glaucoma. The automatic extraction of OD and OC can be achieved by traditional image processing methods and deep learning-based methods.

For traditional image processing methods, OD and OC are detected in the early stages using threshold techniques and morphological operations [3,4]. Hough transform and active contour models were also employed to detect OD and OC in fundus images [5,6,7,8]. In later research, superpixel based methods achieved better local consistency for OD and OC segmentation [9,10]. The above methods mainly used the differences in colors between the inside and outside of the boundary of OD (OC) to determine OD (OC). The information regarding blood vessel bends is in fact also important when an ophthalmologist determines the location of the OC. In the automatic extraction of OC, the blood vessel bends are also used to further improve the segmentation accuracy [8,11]. In another study [12], both the color difference and the vessel bends information were combined to determine the position of OC. However, there is a large difference between the OC obtained by these methods and the OC marked by ophthalmologists.

In recent years, deep learning has developed rapidly in the field of computer vision and has achieved exciting results in image classification [13,14], object detection [15,16] and image segmentation [17,18]. The deep convolutional neural network (CNN) can automatically extract represented features from the input images. CNNs have also achieved a good performance in the segmentation of OD and OC. In [19], a U-shaped CNN was designed to segment OD and OC and an improvement was obtained in comparison with the use of classical methods. In [20], a CNN combined with an ensemble learning technique was proposed. In this method, informative seed points were selected by entropy sampling, and the graph cut algorithm as well as the convex hull transform was used as a post-processing step to obtain the final segmentation. In [21], the multi-label loss function and polar transformation technique were utilized in a U-shaped CNN, which can segment both OD and OC simultaneously.

The segmentation methods of OD and OC based on deep learning are more accurate than classical methods. Deep learning methods have achieved high precision when applied to the task of OD segmentation. In OC segmentation, the OCs extracted by deep learning methods are close to those masked by the glaucoma experts for most fundus images with conventional styles. However, because the above methods used the average loss minimization strategy (ALM) to train the deep network, the prediction accuracy of a few fundus images with special styles was not high enough. In the case where the cup is extremely small or the contrast between the inner and outer areas of the OC outline is not obvious, the OCs extracted by the above deep learning methods have big errors compared to the OCs marked by glaucoma experts.

In order to solve this problem, we designed a U-shaped CNN with multi-scale and multi-kernel module (MSMKU) for OD and OC segmentation. In the proposed multi-kernel module, parallel convolution layers with different convolutional kernels were used so that the model could obtain denser feature information and better capture OC information of different sizes. Furthermore, in order to make accurate predictions for fundus images with special styles, we designed a mixed maximum loss minimization (MMLM) training strategy to train the proposed MSMKU. In this paper, we made the following contributions to the community:

(1) We introduced a fully automatic framework for accurate segmentation of OD and OC. The main network structure is a U-shaped CNN. Compared with the standard U-Net, the multi-scale features were effectively represented, thereby improving the overall accuracy of the segmentation.

(2) We designed the MMLM learning strategy to improve the accuracy of the segmentation model. It can adaptively sort samples by the loss function and re-weigh the samples through data enhancement. This training strategy can synchronously improve the prediction performance of all samples, thus ensuring better generalization performance of the algorithm. This training strategy can be used not only to train the MSMKU proposed in this paper, but also to train other deep neural networks.

(3) The proposed method was validated on two public datasets for OD and OC segmentation. We not only compared the proposed method with the latest methods in this field, but also quantitatively analyzed the relationship between the data distribution and the learning process. All the results are open to the community for further research.

## 2. Related Work

### 2.1. Maximal Loss Minimization Learning Strategy

In the field of machine learning research, a commonly used learning rule is to approximately minimize the average loss, while another option is to approximately minimize the maximal loss. The former learning rule treats each training sample equally when performing parameter optimization, while the latter learning rule only focuses on the training sample with the largest loss when performing parameter optimization. Some classical machine learning algorithms, such as AdaBoost [22] and support vector machines (SVMs) with hard margin [23], can be viewed as approximately minimizing the maximal loss. In some situations, the training samples can be divided into “typical” samples and “rare” samples, and the goal is to have a high accuracy for both typical and rare samples. It has been proven that under some conditions, minimizing even few rare samples was sufficient in guaranteeing a good performance on unseen samples from the rare scenario [24]. The average top-k loss is a natural generalization of the maximal loss, which is the average over the k-largest individual losses over a training dataset [25]. The SVM with average top-k loss obtained a good prediction performance for binary classification and regression on synthetic and real datasets [25].

### 2.2. Deep Learning with Maximal Loss Minimization

In the field of deep learning, maximal loss minimization (MLM) has been successfully used to improve prediction accuracy. An importance sampling scheme was proposed, which is suitable for use with deep learning models [26]. It adopts an optional biased gradient estimator that can focus on hard examples in the training set and improve the generalization performance of prediction models. Furthermore, it was shown that the loss can be approximated with a model with significantly lower complexity. The generality of this method was evaluated on both image classification and language modeling tasks using deep convolutional and recurrent neural networks.

Under the framework of deep learning, the concept of focal loss is similar to maximal loss, and is mainly used in dense object detection [27]. It is designed to address class imbalance by down-weighting inliers (easy examples) such that their contribution to the total loss is small, even if the number of them is large. This method focuses on training using a sparse set of hard examples and realizes the idea by modifying the cross-entropy loss. The proposed method was also designed to address the imbalance problem, not between classes but between typical and rare samples. In addition, the proposed method is for the semantic segmentation task but not the dense object detection task.

## 3. Methods

To segment OD and OC from fundus images, we designed a U-shaped fully convolutional neural network with multi-scale input and multi-kernel. Furthermore, considering that the number of images with special styles (such as containing small optic cups) is small in the OD and OC dataset, which leads to poor prediction of the standard training strategy on such images, we designed an MMLM training strategy. This training strategy adaptively focuses on samples with poor prediction and implements data enhancement on these samples, thus achieving a sufficiently high prediction accuracy for the worst-predicted samples.

**Figure 2:**
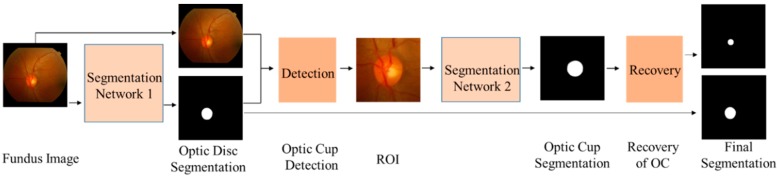
Flow chart of optic disc and optic cup segmentation in two stages.

Considering OC is located inside OD, we first segment the area of OD and then segment OC inside a region containing OD. In this paper, the segmentation of OD and OC were performed in two stages, and two different MSMKUs with the same structure were used for the segmentation of OD and OC, respectively. Figure 2 illustrates the entire processing flow for the proposed segmentation of OD and OC. Firstly, the fundus images were input into the first MSMKU to train a model for the segmentation of OD. Then the obtained masks of OD were used to perform region of interest (ROI) detection and the ROIs of the fundus images were input to the second MSMKU to train a model for segmentation of OC. Finally, OC was restored to their original position in the fundus image based on the coordinates recorded during ROI detection. The two MSMKUs mentioned above were independently trained using the proposed MMLM training strategy. 

### 3.1. U-Shaped Convolutional Neural Network with Multi-Kernel 

**Figure 3:**
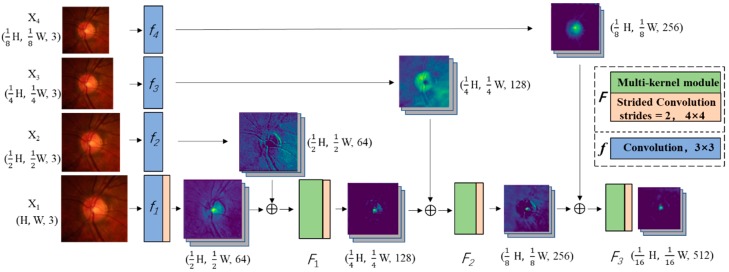
Schematic diagram of feature extraction in the contracting path of optic cup (OC) segmentation.

The proposed MSMKU is based on a U-shaped neural network which contains a contracting path and an expanding path. In the contracting path, fundus images with multiple scales are input to perform feature extraction. Differing from the spatial pyramid pooling module [28], MSMKU uses the image pyramid directly. For OC segmentation, the process of extracting features by the convolutional layers and multi-kernel modules in the contracting path is shown in Figure 3. The process of extracting features for OD segmentation is the same, except that the size of the input image is different. In this process, the input image X_1_ with resolution (W, H, 3) is first resized to smaller images X_2_, X_3_, and X_4_ with resolutions of (W/2, H/2, 3), (W/4, H/4, 3), and (W/8, H/8, 3) respectively. For OD segmentation, W and H are set as 384; for OC segmentation, W and H are set as 256. Each image X_i_ is required to perform convolutional operations at the initial step. Assuming *f*_i_ is the convolutional operations performed on input images X_i_, the output features after the convolution layers can be represented by *f*_i_(X_i_). The size of the output of *f*_i_ is the same as its input; the size of the output of a strided convolution layer is half the size of its input. Assuming *F*_i_ represents the function of the multi-kernel module followed by a strided convolution layer, the output of *F*_i_ is half the size of its input. Then the size of the output of *F*_1_, *F*_2_ and *F*_3_ is (W/4, H/4,), (W/8, H/8,), and (W/16, H/16) respectively. We fused the multi-scale features from different paths by adding them together in a hierarchical way, the output of the contracting path is $F_{3}\left(F_{2}\left(F_{1}\left(f_{1}\left(X_{1}\right)+f_{2}\left(X_{2}\right)\right)+f_{3}\left(X_{3}\right)\right)+f_{4}\left(X_{4}\right)\right)$, and its size is (W/16, H/16).

**Figure 4:**
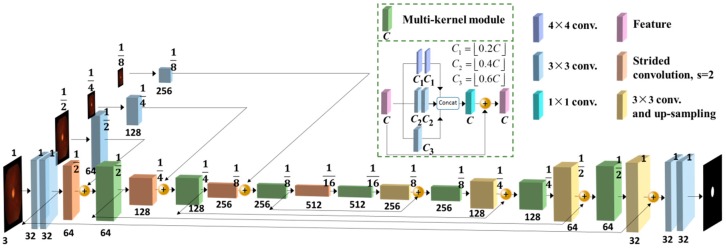
The structure of the U-shaped convolutional neural network with multi-kernel module.

In the contracting network, the role of the convolutional layers and multi-kernel modules is to make a locally weighted combination of the input feature maps to extract more abstract features. In Figure 4, we show a visualization of the features extracted at each convolutional layer and multi-kernel module for a fundus image in contracting path of OC segmentation. In the expanding path, features from the contracting path are added to the correspondent layer to provide low- and mid-level information for up-sampling, and thus obtain full-size segmentation results with sharp details. The whole network of contracting path and expanding path is shown in Figure 4.

Specifically, we made several innovative modifications compared with the original U-Net. Firstly, to maximally maintain the context information in different resolutions, we replaced all of the pooling layers with strided convolution layers. The image pyramid was also used as the input of the network to increase the multiscale context information and receptive field. Secondly, we adopted a multi-kernel module as the basic convolutional module. As illustrated in Figure 4, it contains three 3 × 3 convolution kernels and two successive 4 × 4 convolution kernels to stack the extracted features. After that, a 1 × 1 convolution kernel was used to change the dimension of the features, followed by an RELU activation function and a batch normalization. We also added the short connection surrounding the multi-kernel module for residual correction, which helped to decrease overfitting during the model training. Furthermore, we replaced the original up-convolution layer with the combination of a convolutional layer and a linear up-sampling layer for better feature fusion and detail reconstruction.

### 3.2. Mixed Maximum Loss Minimization Training Strategy

**Figure 5:**
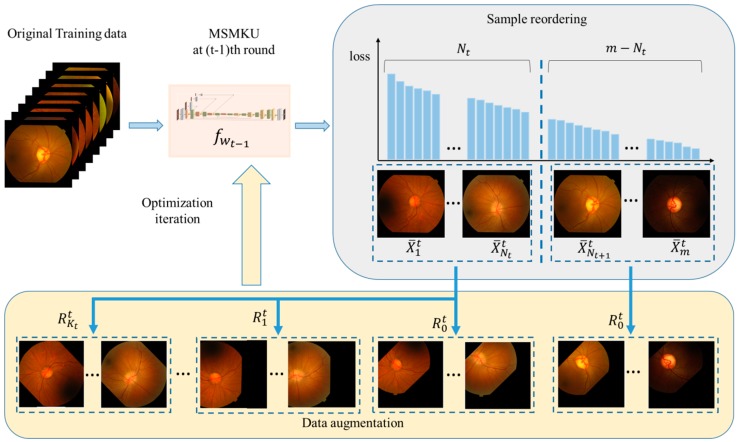
Data augmentation and parameter iteration based on loss sorting.

In the OD and OC segmentation dataset, a small number of images with some specific styles (such as containing a small cup) is usually included, and the prediction accuracy of CNN for these images is not high. One of the main reasons for this is that every image in the training set is used equally in the standard training process of CNN. This leads to the fact that in the early training stage, the prediction accuracy of most images with a conventional style is rapidly improved, but the prediction of a few images with special styles is not accurate enough. In the later stage of training, the prediction accuracy of the images with special styles in the training set is gradually improved, but that of images outside the training set with the same special style is still not high. This suggests that prediction improvement in the later stage of training may be achieved through overfitting. In order to give CNN a better generalization performance, it needs to consistently improve the prediction performance of all the images in the training set during the training process instead of improving the majority in the early stage and then improving the remaining few in the later stage. Based on the above analysis, we designed a training strategy of mixed maximum loss minimization for CNN. According to this training strategy, the loss of each image in the training set was evaluated after each round of parameter iteration. For those images with large losses, data enhancement was performed by random rotation and translation, and the weights of these images were increased in the next round of training.In deep neural network design for image segmentation, the two most commonly used losses are Dice-coefficient (DICE) loss and Intersection over Union (IoU) loss. For the target area G marked by a doctor and the area *P* predicted by an algorithm, the DICE refers to the ratio of the double intersection area of G and P to the sum of their area: $\mathrm{DICE}\left(P,G\right)=\frac{2\left|P\cap^{\;}G\right|}{\left|P\right|+\left|G\right|}$; IoU loss refers to the ratio of the intersection area of G and P to the area of their union: $\mathrm{IoU}\left(P,G\right)=\frac{\left|P\cap^{\;}G\right|}{\left|P\cup^{\;}G\right|}$. In this study, we used the DICE loss, which is concretely calculated as follows:

(1) $$l_{\mathrm{DICE}}[P,G]=1-\frac{2\sum_{i,j}P_{i,j}G_{i,j}}{\sum_{i,j}P_{i,j}^{2}+\sum_{i,j}G_{i,j}^{2}},$$

where *P* represents the probability matrix predicted by the algorithm and *G* represents the binary matrix of ground truth. In the OD (OC) segmentation, $P_{i,j}$ indicates the probability that the network predicts the (*i*, *j*)-th pixel in OD (OC), while $G_{i,j}$ indicates the value of the (*i*, *j*)-th pixel of ground truth where 1 represents inside OD (OC) and 0 represents outside OD (OC). The MMLM training strategy for MKMSU is shown in Algorithm 1. At the *t*-th round of the training, the current total loss $L_{t}$ is defined based on the prediction performance of the prediction function $f_{t-1}$ obtained in the previous round. Here, $L_{t}$ consists of two parts: the first part is the sum of the losses of $f_{t-1}$ on all images in the training set and the second part is the sum of the losses of $f_{t-1}$ on the images with the top $N_{t}$ losses ranked from the largest to smallest in the training set. The losses in the second part contain the losses brought by data enhancement with $K_{t}$ folds. Here, the data enhancement was performed by repeatedly increasing the number of chosen images after random translation or rotation. In Algorithm 1,$\;R_{k}^{t}$ represents the k-th fold data enhancement operation in the t-th round of training and $N_{t}$ and $K_{t}$ are taken as $N_{t}=Max\left\{N_{max}-t,N_{min}\right\}$, and $K_{t}=Max\left\{K_{max}-⎣0.25⎦t,K_{min}\right\}$. Figure 5 visually shows the process of obtaining the images with the first $N_{t}$ largest losses and performing data enhancement with $K_{t}$ folds. Based on the current network parameter $w_{t-1}$ and the total mixed loss $L_{t}$, the parameters are updated by the optimization algorithm “Adam”. In Algorithm 1, $\lambda_{1}$ and $\lambda_{2}$ are used to balance the weight of the two types of losses in the total mixed loss. In the expression of $L_{t}$, if $\lambda_{1}$ is set as 0 and the data enhancement operation in the second term is deleted, then $L_{t}$ degenerates into the top-k maximum loss. Moreover, if $\lambda_{2}$ is set as 0, then $L_{t}$ degenerates into the standard average loss. In addition, in this paper, $N_{t}$ and $K_{t}$ are simply taken as a function that first linearly decreases with the number of rounds of training and then remains constant. In the context of more applications, $N_{t}$ and $K_{t}$ can be set more flexibly based on actual conditions.

**Algorithm 1** Mixed Maximum Loss Minimization Training for MSMKURequire:$\;{\left\{\left(X_{i},Y_{i}\right)\right\}}_{i=1}^{m}$,$\;N_{t},K_{t},\lambda_{1},\lambda_{2}$
Randomly initialize network parameters $w_{0}\;$ and get $f_{w_{0}}$
While w has not converged do
For t = 1, 2, …, 200
Sort $l\left(f_{w_{t-1}}\left(X_{i}\right),Y_{i}\right),i=1,\ldots ,m$ by big to small and get the sample set ${\left\{\left({\bar{X}}_{i}^{t},{\bar{Y}}_{i}^{t}\right)\right\}}_{i=1}^{m}$
$L_{t}$
←
$\lambda_{1}\overset{m}{\underset{i=1}{\sum }}l\left[f_{t-1}\left(X_{i}\right),Y_{j}\right]+\lambda_{2}\overset{K_{t}}{\underset{k=1}{\sum }}\overset{N_{t}}{\underset{j=1}{\sum }}l\left[f_{w_{t-1}}\left(\;R_{k}^{t}\left({\bar{X}}_{j}^{t}\right)\right),R_{k}^{t}\left({\bar{Y}}_{j}^{t}\right)\right]\;$
$w_{t}$
←
$w_{t-1}+10^{-4}\cdot Adam\left(w_{t-1},\nabla L_{t}\right)$
End for
End while 

## 4. Experiments

### 4.1. Datasets and Evaluation Method

We evaluated the prediction performance of the proposed MSMKU with the MMLM training strategy (MSMKU–MMLM) and the compared algorithms on the RIM-ONE-V3 [29] and DRISHTI-GS datasets [30]. The RIM-ONE-V3 dataset contains a total of 159 fundus images, including 85 normal eyes and 74 with glaucoma (including 35 where glaucoma was suspected), each of which was marked by a doctor for OD and OC. For the DRISHTI-GS dataset, we selected 50 fundus images for the evaluation of OD and OC segmentation in [19,20]. In this dataset, OD and OC in each fundus image were marked by four doctors, and the area that was considered as the OD (OC) by at least three doctors was labeled as OD (OC).

In this paper, the *F*-score, value of IoU, sensitivity and specificity were used as evaluation indicators for OD and OC segmentation. In a fundus image, for OD (OC) predicted by an algorithm and OD (OC) marked by the doctor, let TP, FP and FN represent the number of true positive, false positive and false negative pixels, respectively. Define $\mathrm{Precision}=\frac{TP}{TP+FP}\;$and $\mathrm{Recall}=\frac{TP}{TP+FN}$. Then, the *F*-score can be calculated as follows [31]:

(2) $$F=2\times \frac{Precision\times Recall}{Precision+Recall}.$$

And define $\mathrm{IoU}=\frac{TP}{TP+FP+FN}$, $\mathrm{sensitivity}=\frac{TP}{TP+FN}$ , and $\mathrm{specificity}=\frac{TN}{TN+FP}$.

We used the five-fold cross-validation method to evaluate the performance of the proposed algorithm and the compared algorithms. The average *F*-score of the five-fold cross-validation was used as the evaluation indicator. For the RIM-ONE-V3 dataset, the first four folds of data contained 17 normal eyes and 15 glaucoma and the last fold contained 17 normal eyes and 14 with glaucoma. The fundus images of glaucoma and normal eyes were grouped in order of image numbers. For the DRISHTI-GS dataset, 50 fundus images were equally divided into five groups in the order of the image numbers.

### 4.2. Experimental Setup

For the OD segmentation, the original fundus images and the corresponding images of OD marked by the doctors were resized to images with a resolution of 384 × 384 as the input of the OD segmentation networks. For the OC segmentation, the original fundus images and the corresponding images of OC marked by the doctors were first cropped according to the prediction of OD, and then the cropped images were resized to images with a resolution of 256 × 256 as the input of the OC segmentation networks. The proposed basic MSU, MSMKU and MSMKU–MMLM were implemented based on Keras with TensorFlow backend. During training, we employed the Adam algorithm for optimizing the proposed networks. The initial learning rate was set as $10^{-4}$, and other parameters were set as $\beta_{1}=0.9,\beta_{2}=0.999,\varepsilon =10^{-4}$. For MSMKU–MMLM, additional parameters were set as $\lambda_{1}$ = 1, $\lambda_{2}$ = 2, $\;N_{max}$ = 40, $N_{min}$ = 5, $K_{max}$ = 15, $K_{min}$ = 4, $N_{t}=Max\left\{N_{max}-t,N_{min}\right\}$, and $K_{t}=Max\left\{K_{max}-⎣0.25⎦t,K_{min}\right\}$.

At the end of the training for the OD (OC) segmentation, the prediction result of the proposed network was a probability map in which the predicted value of each pixel indicated the probability that this pixel belonged to OD (OC) as predicted by the network. We used 0.5 as the threshold to convert the probability map into a binary map.

### 4.3. Experimental Results

We compared the proposed MSMKU–MMLM with several methods for OD and OC segmentation. The compared methods included classical image segmentation methods (level-set method [32], morphological method [6], superpixel method [9,10]) and seven deep learning methods (small-scale U-Net [19], ensemble CNN [20], FCN [33], SegNet [34], GAN [35], CE-Net [18] and M-Net [21]). The experimental results are shown in Table 1, Table 2 and Table 3. In Table 1, the prediction results of the compared classical image segmentation methods are as reported in [20]. As Table 1 shows, for the OD and OC segmentation, the proposed MSMKU–MMLM obtained much higher *F*-scores than the classical methods on both the RIM-ONE-V3 and DRISHTI-GS datasets.

**Figure 6:**
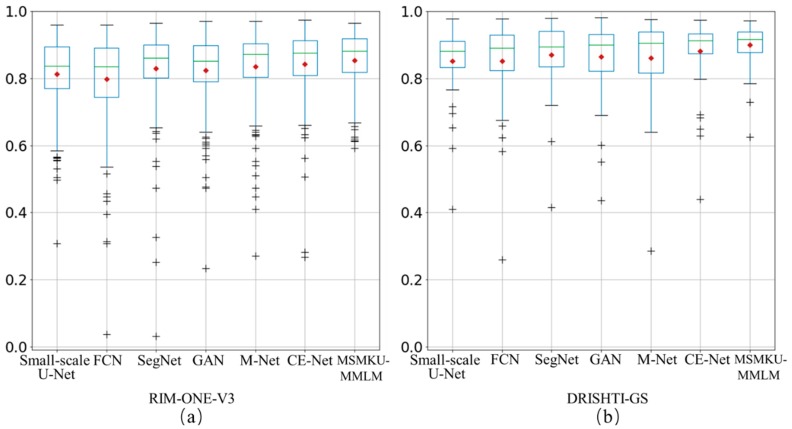
Boxplot of F-score predicted by the proposed and compared methods. (**a**) result on RIM-ONE-V3 dataset (**b**) result on DRISHTI-GS dataset.

Table 2 and Table 3 show the performance comparisons with some deep learning methods on RIM-ONE-V3 dataset and DRISHTI-GS dataset, respectively. In Table 2 and Table 3, the prediction results of Ensemble CNN are as reported in [20]. For both the OD and OC segmentation, the MSMKU–MMLM obtained the highest value for *F*-score, IoU and sensitivity. For OD segmentation, the advantage of the proposed MSMKU–MMLM is not obvious. For the OC segmentation, the *F*-score, value of IoU and sensitivity of the MSMKU–MMLM is significantly higher than the compared deep learning methods; the specificity of the MSMKU–MMLM is almost the same as the compared deep learning methods. Furthermore, Figure 6 shows more details of the *F*-score for MSMKU–MMLM and the compared deep learning methods. As can be seen from Figure 6, the number of points with an *F*-score lower than 0.6 predicted by the MSMKU–MMLM is significantly lower than that of the compared deep learning methods on the RIM-ONE-V3 dataset. The lowest F-score predicted by MSMKU-MMLM is significantly higher than that of the compared deep learning methods on the DRISHTI-GS dataset.

We also evaluated the effect of the two-stage segmentation strategy, multi-kernel network design and MMLM training strategy to improve the segmentation accuracy. Table 4 shows the results of two groups of experiments: the first group uses the ALM to train the basic MSU while segmenting OD and OC jointly (joint MSU), independently (independent MSU) and in two stages (two-stage MSU), as shown in Figure 2; the second group uses ALM, MLM and MMLM to train the MSMKU network to segment OD and OC in two stages (MSMKU–ALM, MSMKU–MLM and MSMKU–MMLM). As can be seen from Table 4, for OD segmentation, the *F*-scores of all methods are roughly the same. For OC segmentation on both the RIM-ONE-V3 and DRISHTI-GS datasets, the two-stage MSU obtained higher *F*-scores than the joint MSU and independent MSU, the MSMKU–ALM and MSMKU–MLM provide a further improvement over the two-stage MSU, and the proposed MSMKU–MMLM obtained the highest *F*-score.

**Figure 7:**
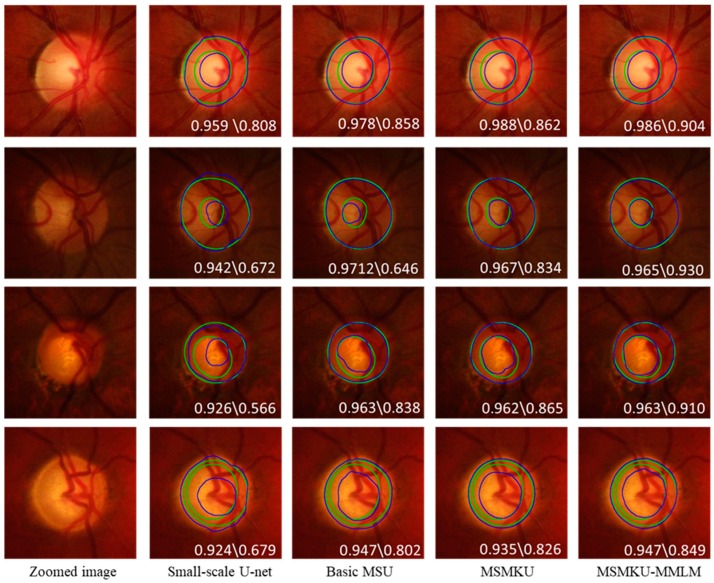
Examples of the OD and OC segmentation by different methods. From the first to fifth column, the examples are zoomed image segmentation results by small-scale U-Net, basic MSU, multi-scale input and multi-kernel modules (MSMKU) and MSMKU–maximum loss minimization learning strategy (MMLM), respectively. The green contours refer to the ground truth, while the blue ones indicate the results of the different segmentation methods. The F-score of OD and OC is shown in each image: F-score of OD\F-score of OC.

In addition, Figure 7 shows the predicted boundary lines of OD and OC by small-scale U-Net, basic MSU, MSMKU and MSMKU–MMLM on four examples of fundus images. In Figure 7, the images on the first two rows are from normal eyes and the images on the last two rows are from eyes with glaucoma.

It can be seen that ODs predicted by all the four methods on the four images were almost the same as the ground truth. However, for the prediction of OC, the prediction of MSMKU–MMLM was much closer to the ground truth than small-scale U-Net, basic MSU and MSMKU.

**Figure 8:**
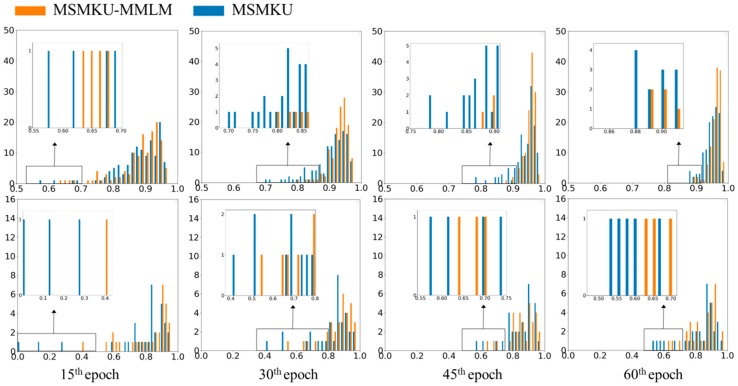
Histograms of the *F*-scores by MSMKU–MMLM and MSMKU on OC in one fold of the RIM-ONE-V3 dataset. The histograms on the first row are results on the training set and the histograms on the second row are the results on the test set. From the first column to the fourth column, the histograms are results trained after 15th, 30th, 45th and 60th epoch, respectively. The orange histograms refer to results by MSMKU–MMLM, while the blue histograms indicate the results by MSMKU. The horizontal axis represents *F*-scores, and the vertical axis represents the number of fundus images.

In order to show more detailed differences between the F-scores obtained by MSMKU–MMLM and MSMKU, we showed the histograms of the F-scores in one-fold cross-validation of the RIM-ONE-V3 dataset (see Figure 8). Considering that the average F-scores of the two training strategies in the training set were no longer improved after 60 epochs, we showed the F-scores after training for 15, 30, 45 and 60 epochs, respectively. From Figure 8, we can see that on both the training set and the test set, the lowest F-scores obtained by MSMKU–MMLM were significantly higher than that obtained by MSMKU. In particular, the training processes of both methods mainly dealt with those easy samples before the epoch 15. After the 15th epoch, both tried to improve the hard samples whose F-scores were in the lower part of the histogram. However, MSMKU–MMLM had a faster convergence than MSMKU alone. The strategy also provides better generalization and a higher accuracy in testing results. After 60 epochs of training (that is, when training was stopped), the lowest four F-scores obtained by MSMKU–MMLM for the test set were all within the interval [0.6, 0.8], while the lowest four F-scores obtained by MSMKU were all lower than 0.6.

**Figure 9:**
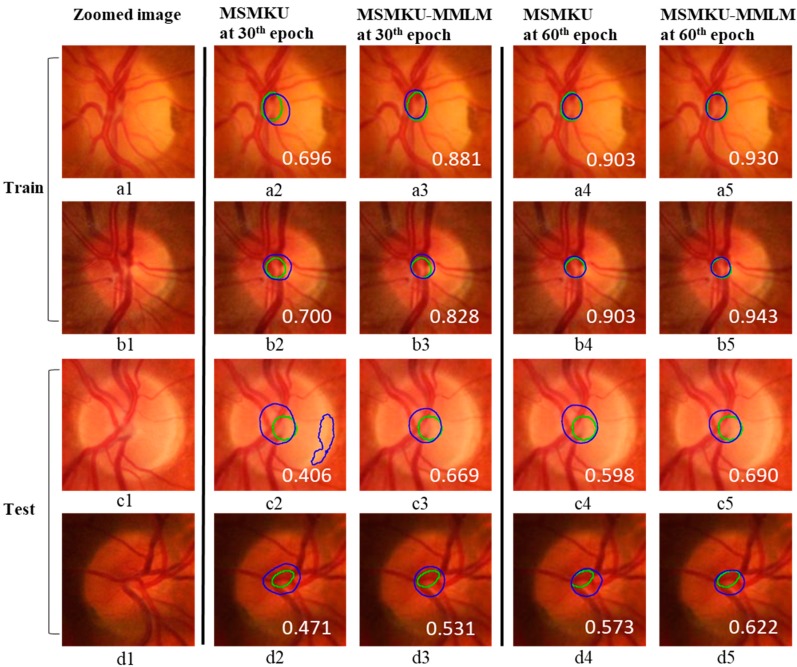
Examples with the smallest *F*-scores of optic cup segmentation by MSMKU–MMLM and MSMKU. The images on the first two rows are results on the training set and the images on the second two rows are the results on the test set. From the first to fifth column, the examples are zoomed image, segmentation results by MSMKU at 30th epoch, MSMKU–MMLM at 30th epoch, MSMKU at 60th epoch, and MSMKU–MMLM at 60th epoch, respectively. The green contours refer to the ground truth while the blue ones indicate the results of the different segmentation methods. The value of F-score of OC is shown in each image.

As shown in Figure 9, we conducted a more detailed analysis for the fundus images with the lowest F-scores by MSMKU–MMLM and MSMKU. In the above mentioned prediction on one-fold of the cross-validation on the RIM-ONE-V3 dataset, after 30 epochs of training, the two fundus images in the training set with the lowest F-scores obtained by MSMKU–MMLM and MSMKU were the same. In the test set, the two fundus images with the lowest F-scores obtained by MSMKU–MMLM and MSMKU were also the same. For these four fundus images, the results of the OC segmentation by MSMKU–MMLM and MSMKU are shown in Figure 9. We can see that these four fundus images were all with small optic cups, and the color contrast between the inner and outer sides of the cup line was not obvious (see a-1, b-1, c-1 and d-1 of Figure 9). On the training set, for MSMKU after 30 epochs of training, there were significant differences between the predicted OCs and the ground truth (see a-2 and b-2 of Figure 9). For MSMKU–MMLM after 30 epochs of training, the predicted OCs were close to the ground truth (see a-3 and b-3 of Figure 9). After being trained for 60 epochs, both MSMKU–MMLM and MSMKU obtained almost the same OCs, as the ground truth for these two cases (see a-4, b4, a-5 and b-5 of Figure 9). 

On the test set, there was a very significant difference between the predicted OCs and the ground truth for MSMKU after being trained for 30 epochs (see c-2 and d-2 of Figure 9), and up to 60 epochs, the difference between the predicted OCs and the ground truth was still very large (see c-4 and d-4 of Figure 9). For MSMKU–MMLM, there was some difference between the predicted OCs and the ground truth after being trained for 30 epochs (see c-3 and d-3 of Figure 9), and after being trained for 60 epochs, the difference was further reduced (see c-5 and d-5 of Figure 9).

**Figure 10:**
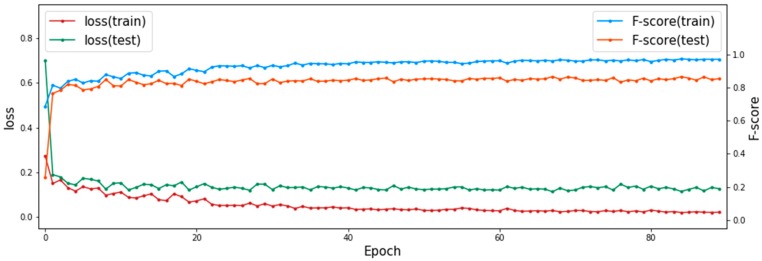
DICE loss and F-score predicted by MSMKU-MMLM at different training iterations.

In addition, in the training process of MSMKU-MMLM on one fold of the RIM-ONE-V3 dataset, the DICE loss and F-score of the training set and test set at different training iterations are shown in Figure 10. It can be seen from Figure 10 that as the DICE loss of the training set decreases, the DICE loss of the test set also decreases; as the F-score of the training set increases, the F-score of the test set also increases. This shows that the model has good generalization performance.

We also evaluated the proposed method for VCDR computation and glaucoma screening by using the calculated VCDR value and ISNT score [36] on the RIM-ONE-V3 and DRISHTI-GS datasets. The RIM-ONE-V3 dataset contains a total of 159 fundus images. After removing 35 cases with unclear diagnosis, there are 85 normal eyes and 39 eyes with glaucoma remaining. Based on the results of OC and OD segmentation of the fundus images, in the test set in 5-fold cross-validation, the VCDR and ISNT score of each fundus image can be calculated. We reported the area under receiver operating characteristic curve (AUC) as an indicator of screening accuracy. The performances for VCDR computation and glaucoma screening are shown in Table 5. The VCDR difference in Table 5 represents the absolute value of the difference between the real VCDR and the VCDR calculated from the predicted OD and OC. The proposed MSMKU–MMLM achieved the smallest VCDR differences and largest AUCs on both the RIM-ONE-V3 and DRISHTI-GS datasets. This result means that the improved accuracy of OC and OD segmentation leads to an increase in the accuracy of glaucoma screening based on the VCDR and ISNT score.

## 5. Conclusions

In this paper, we designed a CNN with multi-kernel module and multi-scale input to segment OD and OC in fundus images. This CNN employed a U-shape network as the body structure. To maximally maintain the information in different resolutions, we constructed an image pyramid to feed multi-level inputs and replaced the pooling layer with a strided convolution layer. Furthermore, a multi-kernel module with short connection was designed to decrease overfitting during the model training. In addition, in order to overcome the problem that the average loss minimization training strategy is not accurate enough for OC segmentation in images with special styles, we proposed a mixed maximum loss minimization training strategy based on data enhancement, called MMLM, which can simultaneously enhance accuracy for all images, thus obtaining better generalization performance. We demonstrated that the proposed MSMKU–MMLM produced state-of-the-art segmentation results on the RIM-ONE-V3 and DRISHTI-GS datasets. Furthermore, the proposed method also obtained satisfactory glaucoma screening performance when using the calculated VCDR and ISNT score on the RIM-ONE-V3 and DRISHTI-GS datasets.

## Figures and Tables

**Table 1:** *F*-score comparisons with classical methods for the segmentation of optic disc (OD) and optic cup (OC).

Methods	RIM-ONE-V3 Dataset		DRISHTI-GS Dataset	
	Optic Disc	Optic Cup	Optic Disc	Optic Cup
Level-set method [32]	0.883	0.726	0.911	0.771
Morphological method [6]	0.901	--	0.932	--
Superpixel method [9]	0.892	0.744	0.921	0.789
Superpixel method [10]	--	0.753	--	0.791
MSMKU–MMLM	0.956	0.856	0.978	0.892

**Table 2:** Comparisons with deep learning methods for OD and OC segmentation on RIM-ONE-V3 dataset.

Methods	Optic Disc				Optic Cup			
	*F*-Score	IoU	Sensitivity	Specificity	*F*-Score	IoU	Sensitivity	Specificity
Ensemble CNN [20]	0.9420	--	--	--	0.8240	--	--	--
Small-scale U-Net [19]	0.9359	0.8808	0.9502	0.9973	0.8128	0.6977	0.7545	0.9976
FCN [33]	0.9508	0.9081	0.9494	0.9984	0.7973	0.6818	0.8011	0.9985
SegNet [34]	0.9483	0.9080	0.9449	0.9985	0.8299	0.7250	0.8081	0.9967
GAN [35]	0.9532	0.9122	09457	0.9987	0.8250	0.7165	0.8142	0.9965
CE-Net [18]	0.9527	0.9115	0.9502	0.9986	0.8435	0.7424	0.8352	0.9970
M-Net [21]	0.9526	0.9114	0.9481	0.9986	0.8348	0.7300	0.8146	0.9967
MSMKU–MMLM	0.9561	0.9172	0.9521	0.9987	0.8564	0.7586	0.8515	0.9971

**Table 3:** Comparisons with deep learning methods for OD and OC segmentation on DRISHTI-GS dataset.

Methods	Optic Disc				Optic Cup			
	*F*-Score	IoU	Sensitivity	Specificity	*F*-Score	IoU	Sensitivity	Specificity
Ensemble CNN [20]	0.9730	--	--	--	0.8710	--	--	--
Small-scale U-Net [19]	0.9043	0.8350	0.9156	0.9969	0.8521	0.7515	0.8476	0.9881
FCN [33]	0.9558	0.9188	0.9611	0.9988	0.8519	0.7590	0.8618	0.9857
SegNet [34]	0.9680	0.9387	0.9652	0.9991	0.8712	0.7836	0.8957	0.9856
GAN [35]	0.9527	0.9185	0.9747	0.9977	0.8643	0.7748	0.8539	0.9907
CE-Net [18]	0.9642	0.9323	0.9759	0.9990	0.8818	0.8006	0.8819	0.9909
M-Net [21]	0.9678	0.9386	0.9711	0.9991	0.8618	0.7730	0.8822	0.9862
MSMKU–MMLM	0.9780	0.9496	0.9792	0.9994	0.8921	0.8232	0.9157	0.9989

**Table 4:** *F*-score comparisons of different training strategies for OD and OC segmentation.

Methods	RIM-ONE-V3 Dataset		DRISHTI-GS Dataset	
	OD	OC	OD	OC
Joint MSU	0.949	0.825	0.974	0.863
Independent MSU	0.952	0.827	0.975	0.869
Two-stage MSU	0.952	0.831	0.975	0.875
MSMKU–MLM	0.953	0.847	0.972	0.884
MSMKU–ALM	0.955	0.849	0.979	0.883
MSMKU–MMLM	0.956	0.856	0.978	0.892

**Table 5:** Vertical cup-to-disc ratio (VCDR) and area under the curve (AUC) Comparisons of different methods.

Methods	RIM-ONE-V3 Dataset		DRISHTI-GS Dataset	
	VCDR Difference	AUC	VCDR Difference	AUC
Small-scale U-Net [19]	0.067	0.832	0.081	0.800
FCN [33]	0.071	0.815	0.091	0.788
SegNet [34]	0.072	0.768	0.079	0.769
GAN [35]	0.063	0.803	0.091	0.748
CE-Net [18]	0.059	0.864	0.076	0.751
M-Net [21]	0.059	0.821	0.092	0.728
MSMKU-MMLM	0.051	0.882	0.054	0.901
